# Nuclear Magnetic Resonance Studies of Bicontinuous Liquid Crystalline Phases of Cubic Symmetry: Transport Properties from ^2^H Nuclear Magnetic Resonance Relaxation Rates

**DOI:** 10.1021/acs.langmuir.3c00825

**Published:** 2023-06-16

**Authors:** Olle Söderman

**Affiliations:** Division of Physical Chemistry, Lund University, P. O. Box 124, SE-22100 Lund, Sweden

## Abstract

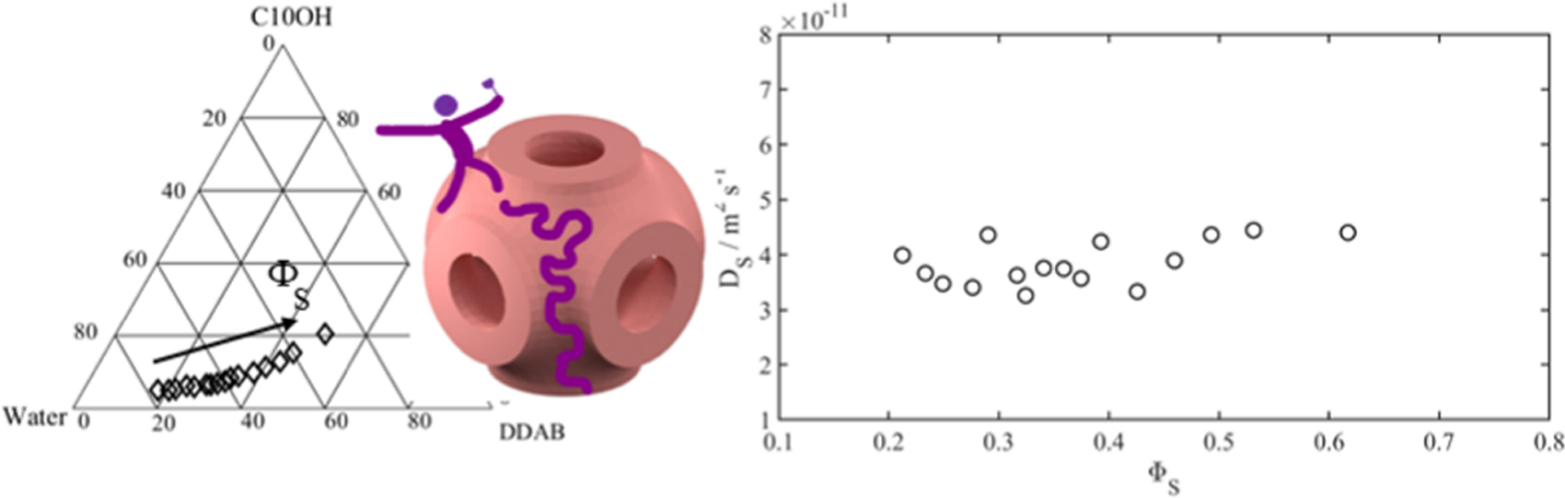

The ternary system didodecyltrimethylammonium bromide, 1-decanol, and water forms an extended reversed continuous phase of cubic symmetry at 25 °C. The cubic phase belongs to the space group *Im*3*m*, as shown by small-angle X-ray experiments. We present extensive deuterium NMR relaxation data from this cubic phase for 1-decanol, deuterated at the carbon adjacent to the hydroxyl carbon position. ^2^H spin-lattice (*R*_1_) and spin–spin (*R*_2_) relaxation rates were measured over the existence region of the cubic phase, which extends from 0.2 to 0.6 in volume fraction of the dividing bilayer surface of the cubic phase. The data are interpreted with an existing theoretical framework for NMR spin relaxation in bicontinuous cubic phases, which takes its starting point in the description of bicontinuous phases using periodic minimal surfaces. Specifically, we obtain the self-diffusion coefficient over the minimal surface in one unit cell for 1-decanol. We also present pulsed field gradient NMR-derived self-diffusion data for didodecyltrimethylammonium bromide and compare the two sets of data. The diffusion data for both components show a mild, if any, dependence on the volume fraction of the bilayer surface. Furthermore, we present diffusion data for the water component in the cubic phase. Finally, we discuss the influences of the choice of the value of the product of the deuterium quadrupole constant and the order parameter *S*. Within the framework of the model used to analyze the relaxation data, a value for this parameter is required. As an initial value, we rely on measurements of deuterium quadrupolar splittings from deuterated decanol in an anisotropic phase.

## Introduction

Lyotropic liquid crystalline phases of cubic symmetry constitute an interesting class of soft materials.^1−4^ They can be formed by low-molecular-weight synthetic surfactants and also by more complex lipid mixtures. Cubic phases can conveniently be divided into discrete and bicontinuous structures. The former is built up of discrete aggregates, often spherical or spheroid micelles with moderate axial ratios. The bicontinuous phases have sample spanning paths in both the polar and nonpolar constituents.

There are several reasons why cubic phases have attracted interest within the scientific community. They are implicated as relevant in many important biological phenomena.^3^ Cubic phases are used in the context of drug delivery, for instance, in the form of nanoparticles, often called cubosomes. The literature on cubosomes has grown extensively^5−9^ since their introduction by Larsson (apparently, the term was first used by Larsson in ref (10)). A case at hand is constituted by cancer treatment, where cubosomes are used to deliver anticancer drugs.^11,12^ Cubic phases can accommodate a wide range of globular proteins that retain their native structure.^13^ Finally, there are outstanding scientific questions pertaining to cubic phase structures and the dynamic state of their constituents.^14,15^ Information on these topics is important in the context of the above-mentioned applications of cubic phases.

NMR spectroscopy has played an important role in studies of cubic phases. One reason for this situation is the fact that NMR spectra of cubic phases resemble those of liquid samples, even though they have very high bulk viscosities. This is sometimes confusing since NMR spectra of solid samples are generally characterized by very broad NMR bands. The narrow NMR peaks from cubic phases are due to the symmetry of the cubic unit cell, which averages relevant static interactions to zero. Thus, information from, e.g., quadrupolar splittings of deuterated components, as obtained in anisotropic liquid crystalline phases, is not available for cubic phases.^16^ On the other hand, all of the NMR machinery for high-resolution NMR spectroscopy can be used.

NMR-derived macroscopic self-diffusion coefficients report directly if a cubic phase is discrete or bicontinuous. An early example is the work of Bull and Lindman that showed that the two cubic phases in the binary dodecyl trimethylammonium chloride (DOTAC)/water system^17^ are discrete and bicontinuous, respectively, since the surfactant self-diffusion coefficients in the two phases differ by 2 orders of magnitude.^18^

The bicontinuous phases have interfaces between their polar and nonpolar parts with complex geometry. The determination of details of the microstructure is sometimes difficult since the preferred technique of X-ray diffraction often yields a limited number of reflections. Here, NMR relaxation studies are promising alternatives.

The relaxation is in part brought about by the diffusion along the (curved) dividing surface. Given a suitable theory for how the NMR relaxation rates couple to the geometry of the surface and the rate with which the studied molecule diffuses along the surface, information on these two quantities may be derived. Earlier work along these lines approximated the geometry of the dividing surface with a sphere inscribed in the cubic unit cell, and relevant NMR relaxation equations developed for spheres were used.^19^ One shortcoming of this rather crude model is that it predicts the same results for samples with equal unit cell sizes for all space groups of cubic phases.

Subsequently, one has come to realize that bicontinuous phases can be successfully modeled by infinite periodic minimal surfaces (IPMS).^20^ These are periodic in three dimensions and free of intersections. In an important contribution, Halle and co-workers developed a theory for NMR relaxation in such structures.^21^ Results are presented for three IPMS commonly found in surfactant/lipid systems (referred to as the P, D, and G (gyroid) surfaces). To account for the finite concentrations of the constituents (polar and nonpolar), results are also presented for the corresponding parallel surfaces in samples with finite volume fractions of water. The theory makes specific predictions for the frequency dependence of NMR relaxation rates and suggests how NMR relaxation data as a function of volume fraction of apolar constituents (for a reversed bicontinuous phase) can be used to distinguish between different IPMS. In a previous report, we have shown that the model developed by Halle et al. can be used to interpret extensive frequency-dependent data from two surfactant systems, and that the parameters derived from the model (order parameters and correlation times) have reasonable values.^22^

**Figure 1:**
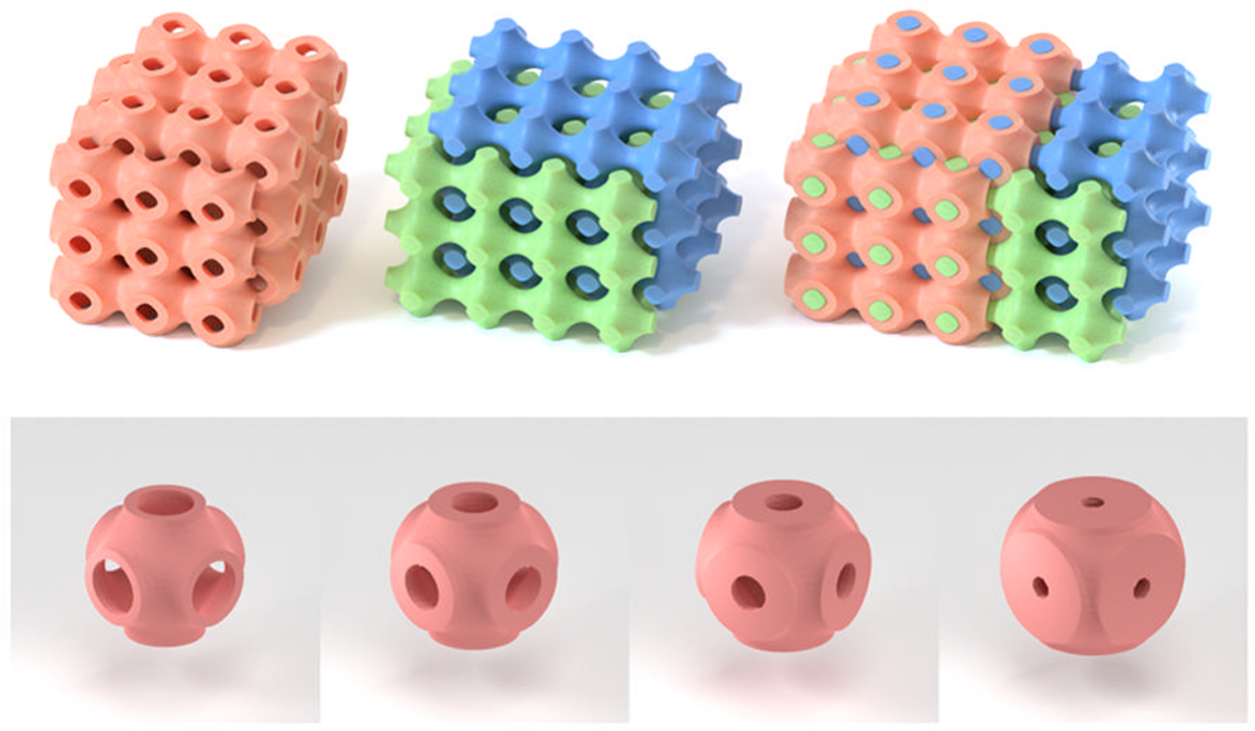
Top: Three-dimensional representation of the cubic phase based on the P-surface. From left to right: the DDAB/1-decanol dividing bilayer, the water channels, and the bilayer and water channels combined. Figure provided by Thomas Meikle (personal communication). Bottom: The unit cell of the P-surface with volume fractions: Φ_s_: 0.2, 0.35, 0.5, and 0.7 from left to right.

In the present study, we apply the model to deuterium relaxation data obtained from 1-decanol, specifically labeled with deuterium in the methylene group adjacent to the carbon carrying the OH group, in a reversed bicontinuous phase formed by didodecyltrimethylammonium bromide (DDAB), 1-decanol, and water. The cubic phase is stable over an extended range of volume fraction of the dividing bilayer surface, which fact makes it possible to examine the predicted model dependence on bilayer volume fraction. In addition, we present NMR PGSE diffusion data for the same system and some SAXS data, the latter used to determine the structure of the cubic phase. A rendition of a reversed cubic phase of the P surface variety, due to Thomas Meikle, is given in Figure 1. From the analysis of the relaxation as well as PGSE experimental data, we obtain self-diffusion coefficients for both amphiphilic components along the surface defined by DDAB/1-decanol bilayer (see the left representation in the top panel of Figure 1) as well as the water diffusion through the water channels (outlined in the middle of the top panel of Figure 1). In Figure 1, we also display the unit cell of the P-surface at different volume fractions of surfactant for a reversed structure.

## Experimental Section

### Material

DDAB was obtained from Tokyo Kasei and was used without further purification. 1-Decanol, deuterated in the carbon adjacent to the hydroxyl carbon, was from Larodan Lipids, Malmö, Sweden. 1-Decanol for the NMR diffusion measurements was from Sigma, with purity better than 98%. All samples were prepared with Milli-Q purified water.

The samples were prepared in glass tubes that were flame-sealed and placed in an oven at 50 °C for 24 h and then allowed to equilibrate at 25 °C for several weeks in the dark. Samples in the cubic phase were optically isotropic when viewed through crossed polarizers. Samples in the lamellar phase were birefringent.

The volume fractions of DDAB + 1-decanol were computed from the densities, obtained from group volumes,^23^ of the components. Values used were: 0.9738, 0.829, 0.839, and 0.99701 g cm^–3^ for DDAB, 1-decanol, 1-αD_2_-decanol, and water, respectively.

### Methods

^2^H NMR measurements were performed at 2.3 T on a Bruker MSL 100 spectrometer. The sealed glass tubes were inserted in 10 mm NMR tubes. The spin-lattice relaxation times *T*_1_ were measured with the inversion-recovery method, using typically 16 delays. The spin–spin relaxation times *T*_2_ were determined from the line widths after corrections for the contributions from magnetic field inhomogeneities. Errors in the ^2^H relaxation data are typically better than ±1.5% for *T*_1_ and ±3% for *T*_2_ based on repeated measurements.

NMR diffusion measurements were performed on a Surrey Medical Imaging Systems, Inc. (England) NMR spectrometer interfaced to a JEOL FX 100 magnet equipped with an external ^2^H-lock. Some additional experiments were carried out on a home-built spectrometer interfaced with a 100 MHz electromagnet, using a quadrupole gradient coil of “in-house” design and construction. The sealed sample tubes were again inserted in NMR tubes of appropriate size. The units producing the field gradient pulses were of “in-house” design and construction. The experiments were carried out using recommended procedures^24^ with either the spin-echo method or the stimulated echo sequence, depending on the value of the spin–spin relaxation times. The gradient strength was varied between 0.03 and 2.7 T m^–1^, and typically 16 values were used. The errors in the diffusion measurements were typically better than ±2.5% based on repeated measurements. All experiments were carried out at 25 °C. The temperature was controlled by variable-temperature units on the spectrometers with an estimated accuracy in the controlled temperature better than ±1 °C. All relaxation and diffusion data were obtained from the experimental raw data using the Optimization Toolbox (v. 8.5) in MATLAB (R2020a).

An additional diffusion measurement was carried out at 25 °C on a Bruker 500 MHz NMR spectrometer using a DIFF-30 probe and a sample prepared in a 5 mm NMR tube. A stimulated echo sequence using bipolar gradient pulses was used with 48 gradient strength values with a maximum of 3 T m^–1^. The raw data from the spectrometer were analyzed with the General NMR Analysis Toolbox (GNAT) from the Manchester NMR methodology group.

The quadrupolar splittings of two samples (containing 2% α-deuterated decanol) in the lamellar phase were determined at 25 °C on the same spectrometer used for the relaxation measurements. The quadrupolar echo technique was used, using recommended procedures.^16^

Small-angle X-ray scattering measurements at 25 °C were performed on a pinhole camera (Ganesha 300 XL, SAXSLAB, Denmark) covering a *q* range of 0.003–2.5 Å^–1^. The scattered intensity profiles have been obtained by circular integration of the two-dimensional intensity profile.

### NMR Theory

Deuterium is a quadrupolar nucleus with spin quantum number I equal to 1. In motional narrowing, the relaxation rates *R*_1_ and *R*_2_ are given by^25^
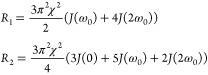
1Here, χ is the quadrupolar coupling constant and ω_0_ is the ^2^H Larmor angular frequency at the magnetic field strength used. The spectral density *J*(ω_0_) is the Fourier transform of the relevant (lab frame) time correlation functions (TCF) *C*_L_(*t*) evaluated at the Larmor frequency ω_0_ (with *k* = 0, 1, and 2)
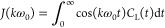
2In order to use eq 1 in the interpretation of relaxation rates from cubic phases, we need to introduce two concepts. First, in surfactant aggregates, motions occur on different timescales. For aggregated surfactant systems, it has proven useful to divide these into two regimes.^26^ There will be librational motions and relatively rapid local motions (such as trans-gauche isomerizations along the hydrocarbon chain). These are assumed to be in the extreme narrowing regime, and hence can be described with an effective correlation time τ_f_. The second dynamic regime is constituted by slower motions, which in the present case are the surfactant diffusion along the curved polar/nonpolar interface over the cubic unit cell. Halle has shown that the contributions of these motions to the NMR relaxation can be decomposed into two components in a model-free way.^27^ Thus, the spectral density *J*(*k*ω_0_) of eq 1 is
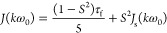
3

The subscripts f and s refer to the fast and slow components, respectively. *S* is an order parameter, quantifying the fraction of the static quadrupolar interaction, which is averaged by the fast, local motions. It is of the same origin as the order parameters derived from quadrupolar splittings in anisotropic phases. By subtracting *R*_1_ from *R*_2_, we eliminate τ_f_ (note: we assume that the fast motions are in extreme narrowing)
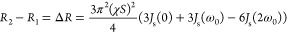
4What remains is to find an expression for *J*_s_(ω_0_). Halle and co-workers^21^ have considered the two irreducible time correlation functions that determine the contribution from surface diffusion in an IPMS bicontinuous cubic unit cell to the NMR relaxation. For a powder sample (where the unit cells are randomly oriented), they show that the relevant lab frame time correlation function is
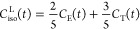
5where the subscript iso refers to the isotropic averaging over the powder sample, and E and T refer to the two irreducible cubic TCFs.

Halle et al. then assume that the two correlation functions on the right-hand side of eq 5 are exponential and justify this with the high rotational symmetry of the cubic unit cell
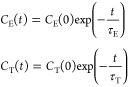
6The two effective correlation times τ_E_ and τ_T_ are different combinations of the surface diffusion coefficient, the average Gaussian curvature, and the fourth rank-order parameter. The resulting correlation function *C*_iso_^L^(*t*) is thus biexponential. To proceed, we note that *C*_iso_^L^ in eqs 5 and 6 is well approximated by a single exponential form
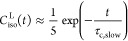
7The definition of the effective correlation time, τ_c,slow_, can be found in ref (21)

**Figure 2:**
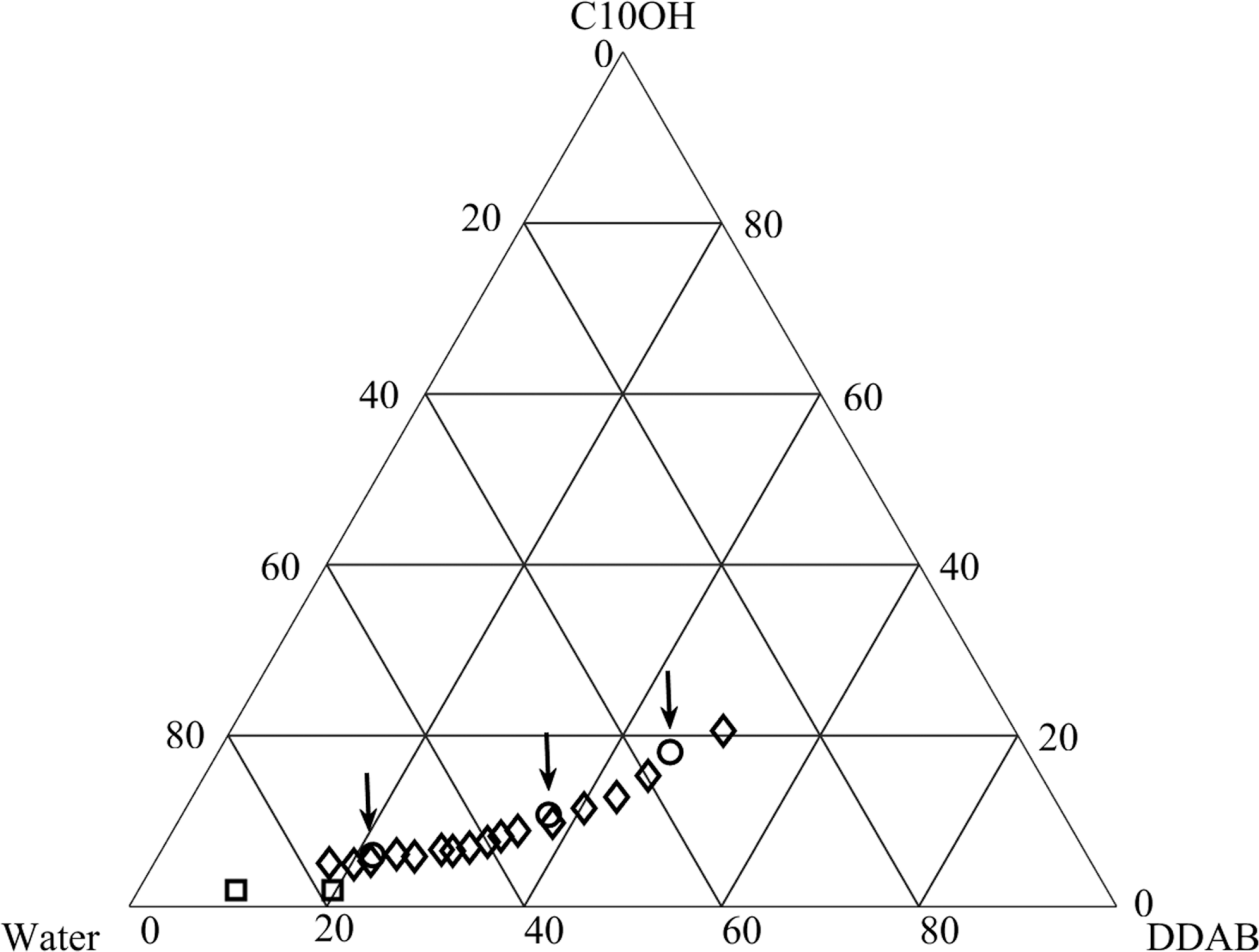
Partial ternary phase diagram of the DDAB/C10OH/water system at 25 °C. Composition of samples studied with NMR is marked with the symbol ◇. Three samples (o), marked with ↓, were subject to SAXS investigations. Finally, the quadrupolar splittings of α-deuterated 1-decanol were measured for two samples in the lamellar phase (□).

In summary, we use the following expressions for the spectral density for the slow motion
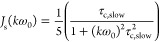
8From eq 4 together with eq 8, we obtain values for τ_c,slow_, provided that the quantity χ*S* is known. This quantity can be obtained from deuterium quadrupolar splittings, Δ, in anisotropic phases. Here, we use splittings in the lamellar phase (see Figure 2), and for this case, . As a point of departure, we take the values from two samples in the lamellar phase formed by DDAB, 1-decanol, and water, but will also discuss variations in the values of χ*S*.

## Results and Discussion

### Background Information on the Phase Diagram DDAB/1-Decanol/Water

The binary phase diagram DDAB/water has been extensively studied. At 20 °C, there are two lamellar phases: one in the concentration range from 4 to 30 wt % surfactant, the second one at considerably higher surfactant concentration (around 80 wt %).^28,29^ Adding a third nonpolar or amphiphilic component results in a rich phase behavior, often dominated by a microemulsion phase emanating from the corner of the added component in the isothermal ternary phase diagram.^28,29^ For some additives, a region of cubic reversed bicontinuous phases emerges at moderate concentrations of additive. Cyclohexane produces two cubic phases,^30^ while styrene gives rise to no less than five specific cubic phases.^31^ Fontell lists several hydrocarbons that produce cubic phases (typically in the range of 5–15 wt %).^32^ Fontell also notes the presence of a cubic phase in the DDAB/1-decanol/water system but gives no further details about the composition and structure of this phase. As far as we are aware, no complete (or partial) phase diagram of the DDAB/1-decanol/water system has hitherto been presented.

In Figure 2, we give the compositions of the samples studied in this work. Cubic phase samples have been found over an extended concentration range (from around 30 to 80 wt %) of water. They are isotropic when viewed through crossed polarizers. As noted above, some compounds produce two or more different cubic phases when added to the DDAB/water system. We did not observe any phase boundaries in our samples; this does not exclude with certainty the presence of more than one cubic phase in the investigated region of the DDAB/1-decanol/water system, as the two-phase regions between two different cubic phases are often quite narrow. Stroem and Anderson show that water diffusion data show discontinuities when passing from one cubic phase to another.^31^ In the present study, we did not observe any such discontinuities (see below under Diffusion data). Finally, we performed X-ray investigations on three samples: at low, intermediate, and high water contents, respectively (indicated by arrows in Figure 2). All three can be indexed to the space group *Im*3*m* (see the SI), corresponding to the P surface. We conclude that the samples studied belong to a single one-phase cubic area.

**Figure 3:**
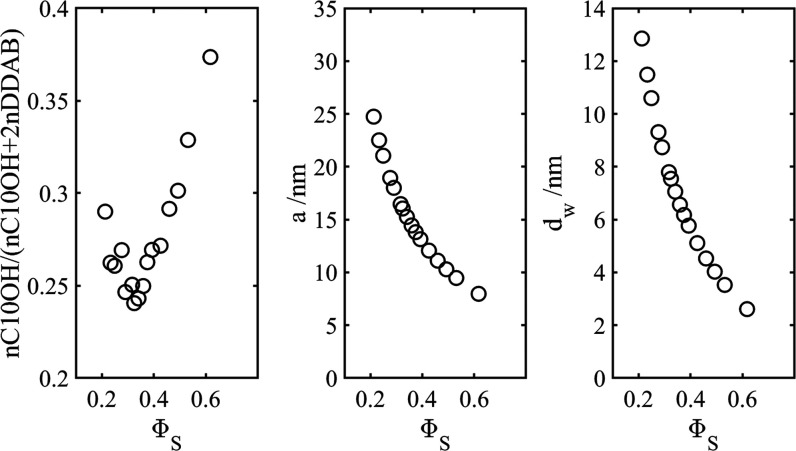
Ratios of 1-decanol hydrocarbon chains to the total number of chains (left), the size of the unit cell (middle), and the thickness of the water channels (right) as a function of the bilayer volume fraction.

In the analysis to follow, we require the unit cell size, *a*, of the cubic phase. This quantity can be obtained from the composition using the following relation:^21^
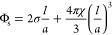
9Here, σ and χ (please note that χ in eq 9 has a different meaning than χ in eq 1) take the values 2.3451 and −4, respectively, for the P surface. Φ_s_ is the volume fraction of surfactant, including the contribution from 1-decanol, which is assumed to reside exclusively in the surfactant bilayer, and  is the thickness of half the bilayer, assigned the value of 11.2 Å for all compositions (see the SI). The ratio of decanol hydrocarbon tails to total the number of tails as well as *a* as a function of Φ_s_ is given in Figure 3. In the same figure, we also give the (average) thickness of the water channel, *d*_w_, calculated from .^33^

**Figure 4:**
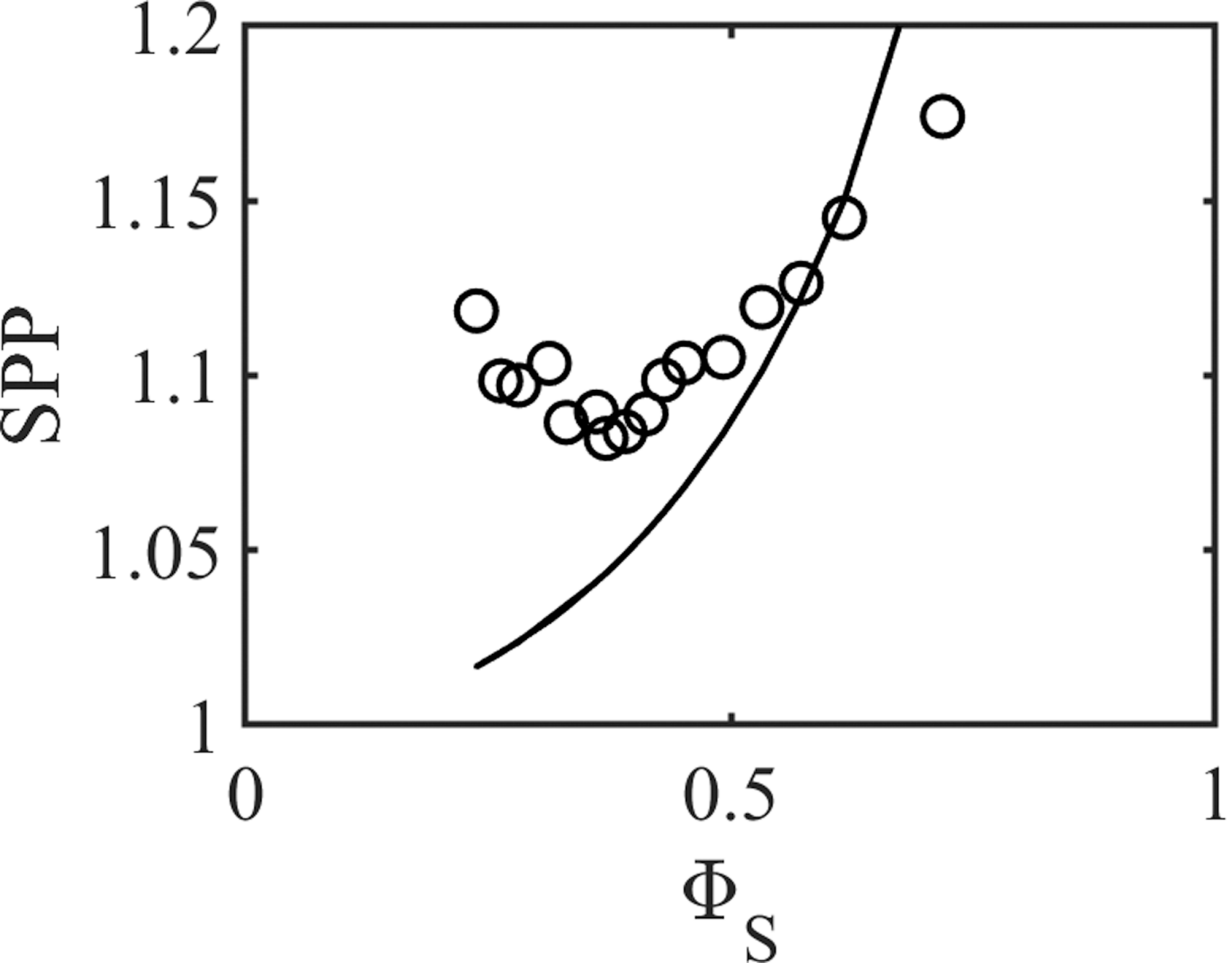
Calculated surfactant packing parameters from sample composition as a function of volume fraction of bilayer (O). The molecular volume of 1-decanol and DDAB were calculated from the densities given in the Experimental Section and headgroup areas were 20 and 75 Å^2^, respectively.  is assigned the value of 11.2 Å. Also given are the predictions based on equations presented in Hyde (see ref (34) for details).

We end this section by noting that the molar ratio of 1-decanol to DDAB increases as the water content decreases (or, equivalently, the volume fraction of bilayer increases), at least for values of Φ_s_ larger than >0.3. This observation can be rationalized by considering the surfactant packing parameter, SPP, , which relates the chain volume per surfactant molecule *v* to the average hydrocarbon length  and headgroup area per molecule, *a*_HG_. Hyde has pointed out that for a reversed bicontinuous phase to be formed, SPP must exceed 1.^34^ Using the results of Hyde, we calculated the SPP for a cubic phase of this category, with space group *Im*3*m* (see caption to Figure 4 for details). The results are presented in Figure 4. As the volume fraction of the bilayer increases, SPP increases. Since both DDAB and 1-decanol reside in the bilayer dividing surface, they can be considered as one effective surfactant. Increasing the relative amount of 1-decanol increases the SPP of the effective surfactant, since the hydrocarbon chain volume *v* increases, while the headgroup area *a* and hydrocarbon chain length  both decrease upon increasing the molar ratio of 1-decanol to DDAB (although the decrease in the latter parameter is marginal, see the SI).

### Note on the Visualization of the Cubic Structure

The dividing surface for the P-surface is often calculated using the relation *F*(*x,y,z*) = cos(*x*) + cos(*y*) + cos(*z*). A more accurate relation for the surface has been presented by Fogden and Lidin: *F*(*x,y,z*) = cos(*x*) + cos(*y*) + cos(*z*) – 0.462 cos(*x*)cos(*y*)cos(*z*).^35^ To depict structures with finite surfactant volume fractions, one typically employs parallel surfaces to the base surface. This is done by constructing two parallel surfaces on either side of the base surface by moving in both directions a distance  along the normal to every point on the base surface. An example pertaining to the present system is shown in Figure 1. Note that the thickness of the surfactant bilayer is the same for all volume fractions, but the size of the unit cell decreases with increasing volume fraction of surfactant.

### Deuterium NMR Relaxation Data

**Figure 5:**
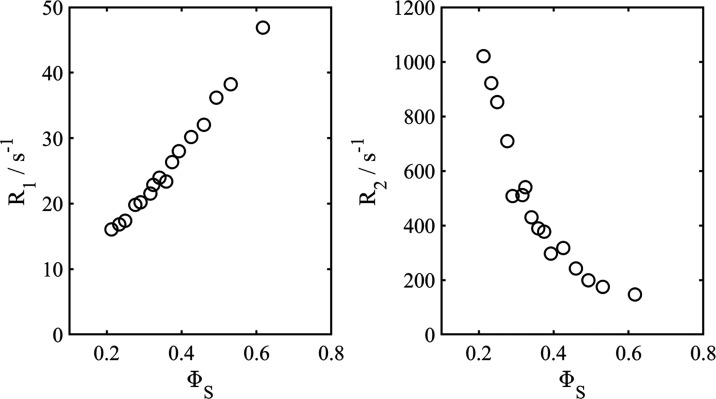
Relaxation rates *R*_1_ and *R*_2_ as a function of the volume fraction of the bilayer. Note the different scales on the y-axis in the panels.

Figure 5 shows the observed deuterium *R*_1_ and *R*_2_ relaxation rates for 1-decanol in the inverted bicontinuous cubic phase in the DDAB/1-decanol/water system at 25 °C, plotted vs the quantity Φ_s_.

Two observations are immediately apparent from Figure 5. First, the values of *R*_1_ and *R*_2_ differ considerably. Second, the dependence of *R*_1_ and *R*_2_ on Φ_s_ is totally opposite. While *R*_1_ increases with Φ_s_, *R*_2_ decreases. With reference to the discussion above on relaxation rates, this implies that the *R*_2_ relaxation is dominated by slow motions (through the zero-frequency spectral density term, see eqs 1 and 3) and that the rate of these motions increases as Φ_s_ increases (or conversely, decrease with increasing lattice parameters). For *R*_1_, on the other hand, the contribution from the slow motion decreases with decreasing Φ_s_, and at high values of the lattice parameter, the fast motion dominates the *R*_1_ relaxation.

**Figure 6:**
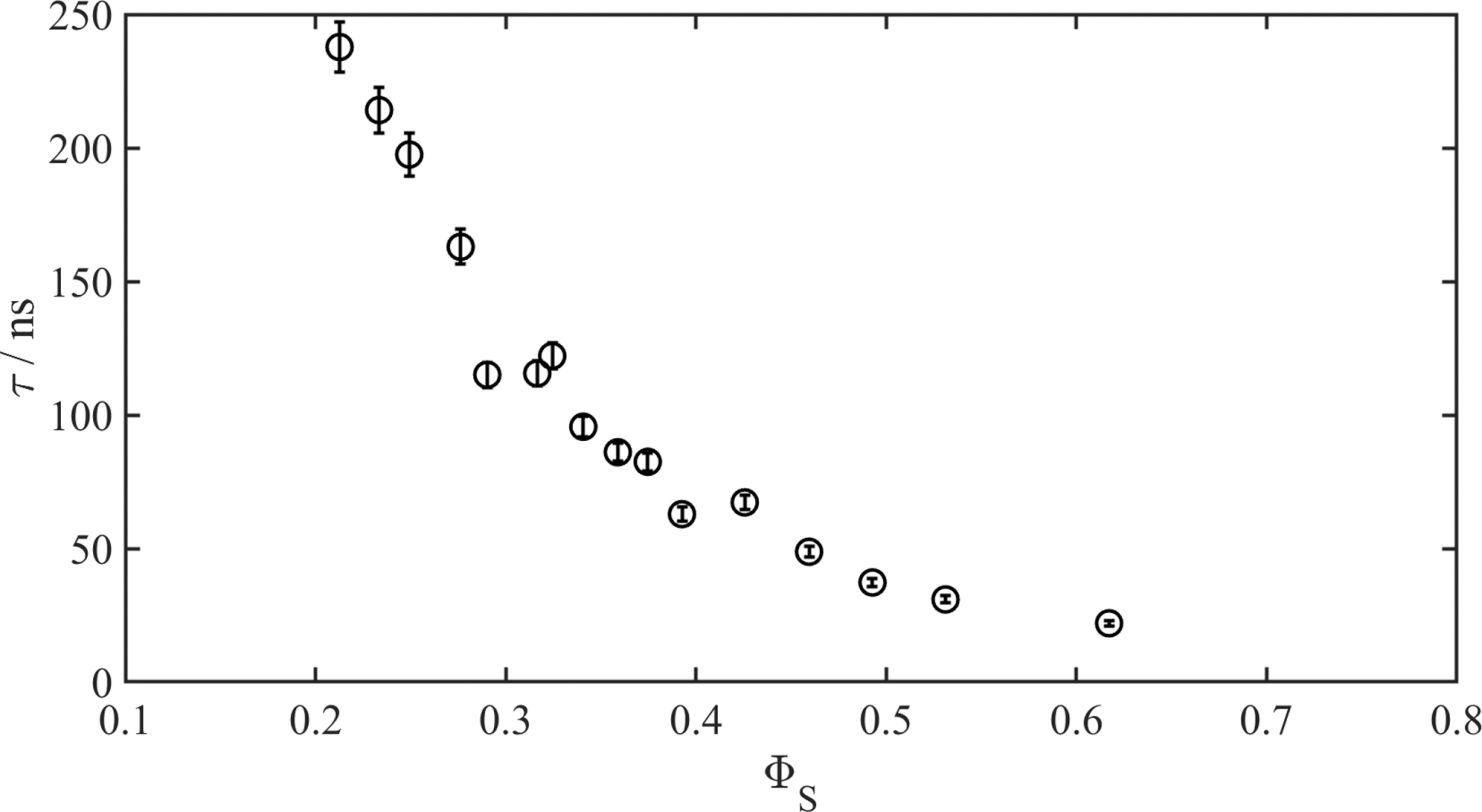
Slow correlation time as a function of volume fraction of bilayer (see text for details). Error bars are calculated from the errors in the measured relaxation rates.

We proceed to calculate the diffusion coefficient for the 1-decanol lateral diffusion over the dividing bilayer in the unit cell. First, we calculate the slow correlation time by combining eqs (4) and (8) using a value for χ*S* =30.83 kHz, which is the average calculated from the quadrupolar splittings measured in the lamellar phase (see Figure 2). The slow correlation times are displayed in Figure 6 as a function of bilayer volume fraction.

**Figure 7:**
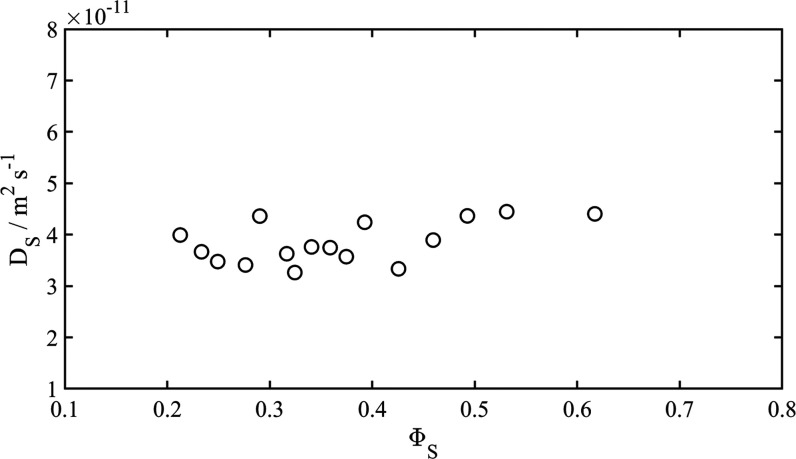
Lateral diffusion coefficients *D*_s_ along the dividing surface for 1-decanol as a function of bilayer volume fraction.

The diffusion coefficient for 1-decanol *D*_s_ is then obtained from the results in ref (21). For the space group *Im*3*m*, the relevant equation is
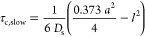
10with *a* the unit cell size (obtained from eq 9 above) and  the monolayer thickness (set to 11.2 Å, see the SI). The results are presented in Figure 7.

We first note that the diffusion coefficient *D*_s_ shows little dependency on the volume fraction of the bilayer. A global fit using eq 10 and assuming a constant value of *D*_s_ yields *D*_s_ = 3.75 ± 0.11 × 10^–11^ m^2^ s^–1^. Within the framework of the relaxation model used, the only parameter value associated with some uncertainty is the value of χ*S*. The value used here corresponds to an order parameter *S* = 0.17 (with χ = 181 kHz^36^), which is a reasonable value. On the other hand, the value of *D*_s_ depends on the square of the order parameter. An increase of *S* by 25% to *S* = 0.21 increases the residual interaction constant χ*S* by around 50%. We will return to this issue below. Finally, we note that the value of the fast correlation time for the C–D vector in 1-decanol can be estimated from the *R*_1_ value obtained for the lowest value of Φ_s_, where *R*_1_ is dominated by the fast motion. The value obtained is 7 ps.

### NMR Pulsed Field Gradient Measurements

The translational diffusion coefficient can also be measured with pulsed field gradient (PFG) NMR. There are, however, two differences compared to the relaxation approach. First, in the NMR PFG experiment, the length scale over which the diffusion is measured is in the μm regime, and thus the diffusion over many unit cells (in the present case a few 100 unit cells) is obtained. In the relaxation measurements, the diffusion is measured over one unit cell. Thus, defects or dislocations in the crystal structure, if present, affect the two approaches differently. Second, the value *D*_obs_ from the NMR PFG approach is obtained from the mean square displacement in the laboratory frame (corresponding to the through-space diffusion coefficient), and thus it is influenced by the fact that the surfactant must follow the dividing surface with a curvilinear diffusion coefficient *D*_s_. The ratio between the two diffusion coefficients is usually discussed in terms of an obstruction effect, β. We will return to this issue below.

**Figure 8:**
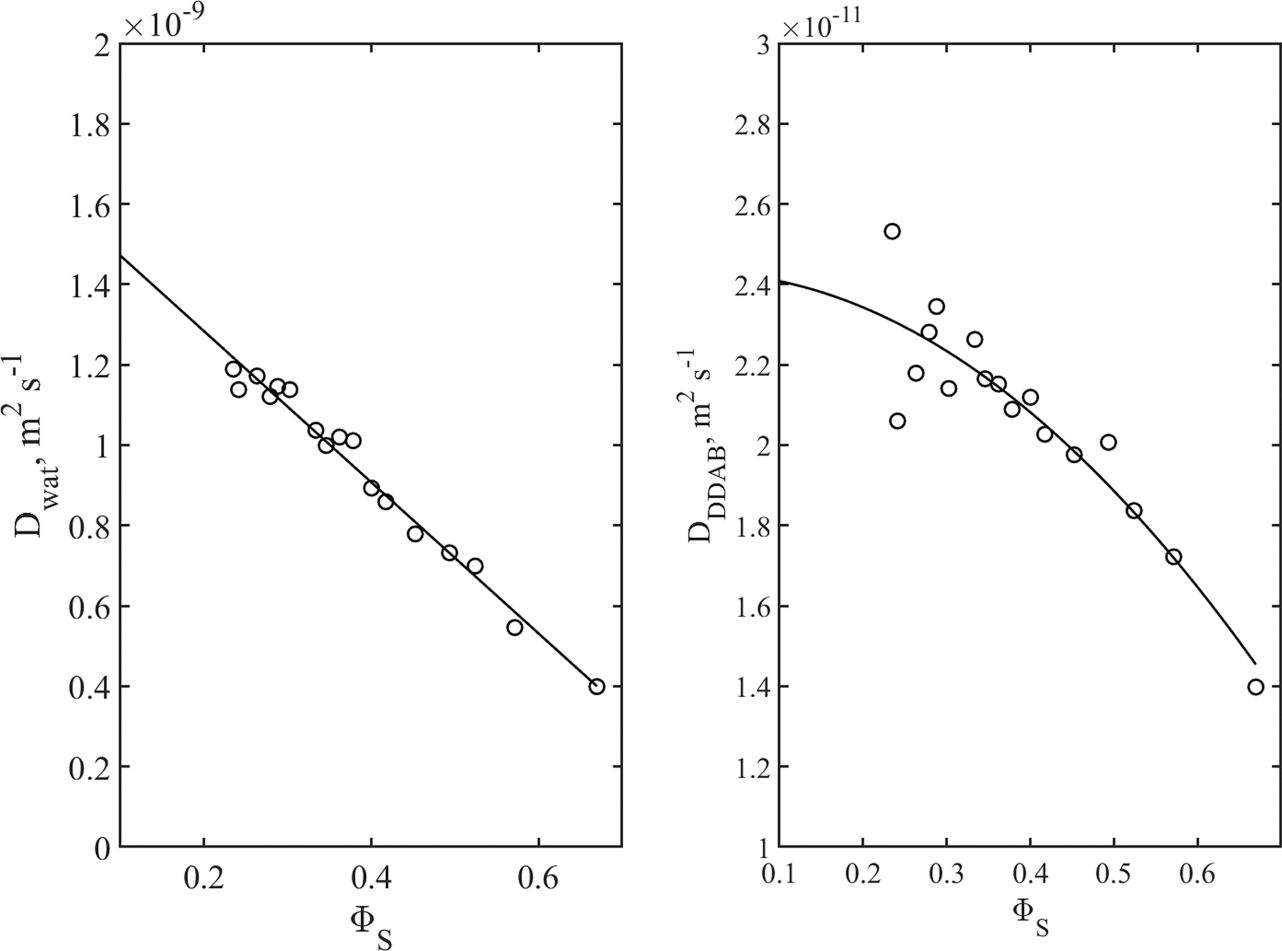
Water (left) and DDAB (right) diffusion coefficients at 25 °C as a function of bilayer volume fraction. The solid lines are the results of fitting eqs 11 and 12 to the water and DDAB data, respectively.

In principle, it is possible to determine the diffusion coefficients of all three components with the NMR PFG approach. At the low field strength used here (2.3 T) and the rather poor resolution on account of the use of sealed glass tubes inserted into NMR tubes, it was not possible to measure the 1-decanol diffusion since the peaks overlap with those of DDAB (the two diffusion coefficients are so close in value that biexponential fits to overlapping peaks do not give accurate results). DDAB, on the other hand, has an isolated peak from the three methylene groups in the headgroup region, and thus the diffusion coefficients of DDAB and, in addition, water can be accurately determined. The results for DDAB and water are presented in Figure 8 as a function of the bilayer volume fraction.

Referring to Figure 8, there is a dependence of the surfactant and water diffusion coefficients on the bilayer volume fraction. Following Anderson and Wennerström^37^ (below referred to as AW) we analyzed the data as follows. For the water diffusion, we fitted the following equation to the data
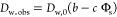
11where *D*_w.0_ is the bulk diffusion coefficient of water at the relevant temperature. Here, we used *D*_w.0_ = 2.2952 × 10^–9^ m^2^ s^–1^.^38^ The values obtained for *b* and *c* are 0.723 ± 0.018 and 0.819 ± 0.046, respectively. Anderson and Wennerström showed that *b* is approximately 2/3 in agreement with the result obtained here. The value of *c* depends on which TPMS one chooses in order to describe the cubic phase.

**Figure 9:**
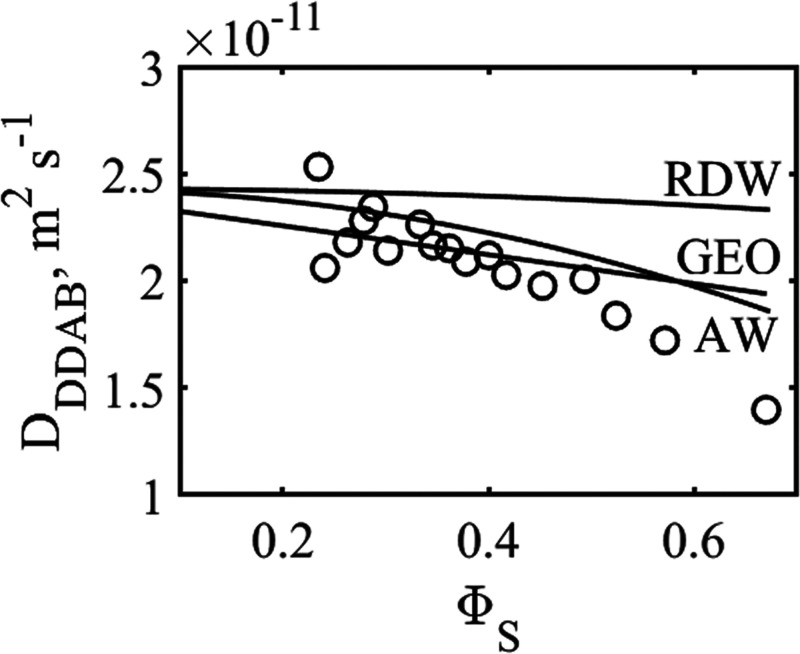
Predicted dependence of the surfactant diffusion on the bilayer volume fraction using three models. AW is the approach of Anderson and Wennerström, RDW is based on random walk simulations of Håkansson and Westlund,^45^ while GEO are the results of the geodesic approach used in the present study. Also shown are the experimental data of Figure 8 (bottom).

For the surfactant case, we use the equation
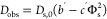
12where *D*_s.0_ is the surfactant curvilinear diffusion coefficient in the limit of Φ_s_ =0. AW analytically proved that *b’* is 2/3 (independent of the TPMS family). We obtained *D*_s,0_ = (3.65 ± 0.10) × 10^–11^ m^2^s^–1^ and *c* = 0.596 ± 0.094, and thus the curvilinear DDAB diffusion along the dividing surface at infinite dilution is (3.65 ± 0.10) × 10^–11^ m^2^s^–1^. AW quoted a value of *c*′ = 0.45 for the P-family of surfaces. We obtained a slightly higher value, and a relevant question is whether there is a concentration dependence in the DDAB curvilinear diffusion, not observed for 1-decanol using the analysis described above (see Figure 7).^39^ AW treated the problem of diffusion in the TPMS systems by solving the relevant partial differential equations for surface and bulk diffusion. To take the bilayer volume fraction into account, they constructed surfaces of constant mean curvature. Other investigators have used random walk simulations on parallel surfaces, using approximate equations for the base minimal surface.^40,41^ Both approaches are fairly involved and require access to high-performance computing facilities. In the present study, we took an alternative approach and calculated the average geodesic distance over the cubic unit cell as well as the average distance through space. We made use of the fact that we were in the long-time limit for the diffusion and so placed a number of points on the surface of the base or the parallel surface of the unit cell using the approximate relation without the improvement suggested by Fogden and Lidin (see above) for the P-surface. We then randomly picked two of these points and calculated the geodesic distance using MATLAB code based on ref (42) as well as the through-space distances (using the algebraic distance formula between two points) and repeated the process. The known values of these quantities for the sphere were used to estimate the number of repetitions needed to get accurate data.^43,44^ On a standard laptop computer, these calculations typically take less than 1 h for each value of Φ_s_. In Figure 9, we summarize the predicted data for the dependence on the volume fraction of *D*_s_.

All three approaches predict a rather mild dependence of the through-space diffusion coefficient for DDAB, with the results in AW giving the strongest dependence. It should be noted that AW used surfaces of constant curvature to predict the dependence, while the two other approaches use parallel surfaces. As noted by Håkansson and Westlund, the approach using constant curvature predicts a larger concentration dependence than random walk simulations on parallel surfaces.^45^ The experimental data display a somewhat stronger concentration dependence than the predicted ones, but the effect is not large. After all, the molar ratio of 1-decanol to DDAB varies with Φ_s_, which fact is expected to influence the DDAB diffusion coefficient. Also, the interaction between surfactants in adjacent layers may play a role, more so for DDAB than for 1-decanol. In fact, the size of the “throats” is quite small at high volume fractions of surfactants (see Figure 1 (bottom) and Figure 3 (right)). It should be stressed that we are determining the lateral diffusion along the bilayer for DDAB. For the water, the diffusion is measured along the water channels, following the dividing surface. Due to the time scale of the measurements coupled to the diffusion paths set up by the composition of the samples, there is no contribution from motion perpendicular to the dividing surface. See also the results from a Brownian dynamics simulation study.^14^

There is one more observation in the comparison of the 1-decanol and DDAB diffusion coefficients that deserves some comments. In summary, we have found for the curvilinear diffusion coefficients in the limit of high dilution of the bilayer 3.85 × 10^–11^ and 3.65 × 10^–11^ m^2^s^–1^ for 1-decanol and DDAB, respectively. This is a surprisingly small difference since DDAB is a twin-chained surfactant.^46^

To shed some light on this issue, we have carried out a very accurate PFG NMR experiment at 500 MHz on a sample with Φ_s_ = 0.3. At this field strength, there is an isolated peak from 1-decanol, and the experiment was optimized for determining the ratio of the diffusion coefficients of 1-decanol to DDAB. The experiment gave a factor of 2.00 for this ratio, implying that the *D*_s_ values for 1-decanol in Figure 7 are in fact too low, and should be on the order of 50% larger. As noted above, within the model, the only input parameter whose value is uncertain is χ*S*. Changing *S* from 0.17 to 0.21 (with χ = 181 kHz) and, as above, performing a global fit to eq 10 yields *D*_s_ = 5.72 ± 0.18 × 10^–11^ m^2^ s^–1^. We assume that χ*S* is constant and does not depend on the ratio of 1-decanol to DDAB. A few % change in *S* as this ratio increases would produce a mild dependence of *D*_s_ on volume fraction, in line with the observation for DDAB.

## Conclusions

In this work, we have presented the surface diffusion of 1-decanol along the surfactant/water interface in a ternary reversed cubic phase and have shown that this value depends marginally on the volume fraction of the dividing surface. We have also presented the diffusion coefficient of the main amphiphilic component and water of the cubic phase using the NMR Pulsed Field Gradient method and have pointed out the differences in the two approaches. While the former reports on the diffusion in one unit cell, the latter measures diffusion over many unit cells. The relaxation model yields the curvilinear diffusion along the dividing surface, while the Pulsed Field Gradient method yields a laboratory-based diffusion coefficient and hence must be corrected for obstruction effects in order to obtain the surface diffusion.

The diffusion coefficient of the components in bicontinuous reversed cubic phases is an important dynamic parameter in the context of the rapidly growing field of applications of cubic phases, for instance, in the context of drug delivery. It is our hope that the methods presented here will be useful in this context.

## Supporting Information Available

Analysis of SAXS data and computation of bilayer thickness (PDF)
