# Supplementary material for: Nuclear Magnetic Resonance Studies of Bicontinuous Liquid Crystalline Phases of Cubic Symmetry: Transport Properties from 2H Nuclear Magnetic Resonance Relaxation Rates
Source: Langmuir. 2023 Jun 16;39(26):9085–93. doi: 10.1021/acs.langmuir.3c00825 (PMC10324400; doi:10.1021/acs.langmuir.3c00825)
Supplement: Supplementary file 1 — la3c00825_si_001.pdf [file la3c00825_si_001.pdf]

Supporting information.

Nuclear Magnetic Resonance Studies of Bicontinuous Liquid Crystalline Phases of Cubic Symmetry.

Transport Properties from ^2H Nuclear Magnetic Resonance Relaxation Rates.

*Olle Söderman**

Division of Physical Chemistry, Lund University, PO Box 124, SE-22100 Lund, Sweden

Analysis of Small Angle X-ray Scattering Experiments.

To determine the space group of the cubic phase presently studied, SAXS experiments were carried out on three samples. The q-values obtained from the diffractograms (see Experimental for details) were fitted to the relation:

$$q = \frac{2\pi\left(h^2 + k^2 + l^2\right)^{1/2}}{a} \qquad \text{(S1)}$$

The data for all three samples could be indexed to values of $\left(h^2 + k^2 + l^2\right)^{1/2}$ from the series $\sqrt{2} : \sqrt{4} : \sqrt{6} : \sqrt{10} : \sqrt{12} : \sqrt{14} : \sqrt{16} : \sqrt{18}$, which is valid for the *Im3m* space group.

The results of the analysis are summarized in Table S1.

Table S1. Results from analysis of SAXS experiments at 25 °C.

Sample	Φ_S	Number of diffractions analyzed	a / Å
1	0.65	6	82 ±1
2	0.49	6	119 ± 2
3	0.29	3	176 ± 6

Calculation of the thickness of the dividing bilayer surface.

In the analysis of the relaxation data, we require a value for the thickness of the dividing bilayer surface. The value used was obtained in the following manner. The length of a stretched hydrocarbon chain of n_C carbons (in nm) is given by:

$$L = 1.5 + 1.27\, n_C \qquad \text{(S2)}$$

The thickness of a DDAB bilayer is 2.4 nm.[1] Hence the thickness corresponds to 70 % of two stretched chains. In terms of the fraction of decanol hydrocarbon chains, the composition of the dividing surface varies from 0.25 to 0.38 (see Figure 3). Taking a value of 1/3 for the fraction of decanol yields an effective hydrocarbon chain of 11.3 carbons and a bilayer thickness (with 70 % of two stretched chains) of 2.23 nm, or 1.12 nm for the monolayer, which is the value used in the analysis of the relaxation data.

References:

(1) Dubois, M.; Zemb, T. Phase Behavior and Scattering of Double-Chain Surfactants in Diluted Aqueous Solutions. *Langmuir* **1991**, *7* (7), 1352–1360. https://doi.org/10.1021/la00055a011.
